# Seven draft genomes for halophilic bacteria from samples in Northern California

**DOI:** 10.1128/mra.00192-25

**Published:** 2026-01-30

**Authors:** Hannah Starcevich, Kush Narang, Abhineet Ram, Sinead C. Archdeacon, Luna Browne, Lily Chen, Avni Duda, Alexander Guess, Zachary Hom, Claire Hsieh, Jion Hwang, Tanish Iyer, Vishista Jain, Emily Jarvinen, Karen Kee, Guadalupe Lauro, Bao Ngoc Le, Gabrielle Lewis, Yicheng Lou, Tien Ly, Rhea Manjunath, Li Meinhold, Adam Mhassen, Adyasha Padhi, Erick Quintana, Cara Razma, Natalya Rosental, Johnnie Runyon, Madison Sparks, Jonathan Sunkari, Joanne Szeto, Chloe Tannous, Camila Sanudo Thomas, Rafael Vasquez-Chan, Luis Lopez Vera, Alexis Vlavianos, Ariel Wang, Sophia Warnock, Zifeng Xu, Taixiang Yang, Rui Yu, Samuel Zaki, Devan Murphy, Carolyn K. Teragawa, Andrew I. Yao, John G. Albeck, Jessica Lumian, Alex S. Nord, William Hemstrom

**Affiliations:** 1 Microbiology and Molecular Genetics, University of California 8789, Davis, California, USA; 2 College of Biological Sciences, University of California 117238, Davis, California, USA; 3 Department of Molecular and Cellular Biology, University of California 240437, Davis, California, USA; 4 Genome Center and Biomedical Engineering, University of California 8789, Davis, California, USA; 5 Department of Earth and Planetary Sciences, University of California 8789, Davis, California, USA; 6 Department of Evolution and Ecology, University of California 166622, Davis, California, USA; 7 Department of Biology, Colorado State University 3447, Fort Collins, Colorado, USA; The University of Arizona, Tucson, Arizona, USA

**Keywords:** halophiles, genomes

## Abstract

Halophiles, extremophiles that live in high-salinity environments, are critical to a range of industrial and biotechnical processes but are relatively understudied. To help build a better understanding of halophilic genomes, we sequenced the genomes of seven halophilic bacterial species from Northern California.

## ANNOUNCEMENT

Halophiles are extremophilic organisms that thrive in high-salinity environments. To survive and maintain enzymatic activity in the presence of high salt concentrations, halophiles have evolved distinct molecular and cellular adaptations (1). Certain bacterial strains already have applications in the food industry (e.g., production of kimchi, pickles, and soy sauce in the food industry) (2). Additionally, researchers continue to discover new functions for these bacterial enzymes that suggest increasingly important roles in critical industrial and biotechnological processes, including biofuel and bioplastic production (3).

As part of an undergraduate course, students isolated and cultured microbes obtained from several high-salinity environmental samples from Davis, CA, USA, and nearby coastal areas (Table 1). In each case, samples were collected directly from the substrate surface. Approximately 0.1–0.5 mL of substrate was collected in each instance. To isolate a range of halotolerant strains, samples were inoculated in liquid *Halobacterium* media (DSMZ medium 372; see https://www.dsmz.de/microorganisms/medium/pdf/DSMZ_Medium372.pdf) (4) with either 150 or 200 g NaCl per 1,000 mL of media. Samples were incubated aerobically on a shaker at 37°C for 7 days. Turbid cultures were transferred to solid 372 media at the same NaCl concentrations by spread. Pure liquid cultures were then grown by inoculating media with isolated colonies and incubated again as above in the same media as before. Purity was assessed by visual analysis of streak-plated pure cultures and microscopy.

**TABLE 1:** Sample name and origin, number of reads and millions of bases sequenced, total assembly size, number of contigs, N50, L50, % GC content, closest 16S BLAST taxonomy match and percent identity, percent and count of estimated salt-tolerant proteins, and SRA accession numbers for the non-contaminated whole-genome assemblies

Sample name	Sample origin	No. of reads and mBP	Total size (bp)	No. of contigs	N50	L50	GC%	Closest taxonomy match	% Identity	% Predicted salt-tolerant proteins (count)	SRA accession
B23F22_5_S4	Dry soil	1,071,283 and 178.3	4,131,604	49	326,971	5	42.46	*Halobacillus* sp. strain B23F22_5	100.00	9.8 (400)	SRS22781033
B23F22_20_s10	Sandy mud	885,210 and 147	4,653,660	353	168,352	10	65.14	*Halomonas* sp. strain B23F22_20	98.70	24.5 (1,013)	SRS22781034
B23F22_3_S3	Sandy mud	769,699 and 128	3,876,936	16	604,382	3	68.03	*Halomonas* sp. strain B23F22_3	99.90	26.9 (953)	SRS22781031
B23F22-10	Frying pan	1,204,673 and 197	3,475,311	12	575,820	3	68.21	*Halomonas* sp. strain B23F22_10	99.90	30.8 (976)	SRS22781030
B23F22_1_S1	Sand	1,556,771 and 250.5	4,144,087	13	1,406,929	2	40.44	*Halobacillus* sp. strain B23F22_1	98.90	12.8 (527)	SRS22781029
B23F22_16_S9	Dry soil	1,228,607 and 202.8	3,951,710	56	130,875	10	42.96	*Thalossobacillus* sp. strain B23F22_16	99.70	10.8 (419)	SRS22781028
231237_7MaSpsaltlick	Cow salt lick	822,916 and 399.4	6,414,125	326	302,755	7	32.69	*Staphylococcus* sp. strain 231237_7MaSpsaltlick	100.00	4.2 (261)	SRS23575387

Genomic DNA was isolated from liquid cultures using either Wizard Genomic DNA Purification (Promega, Madison, WI, USA) or Monarch DNA extraction (New England Biolab) kits. Whole-genome shotgun libraries were prepared by the University of California, Davis Genome Center, or via MicrobesNG. In the former case, reads were first sheared, then converted to sequencing libraries using a Kapa Hyper Library Preparation Kit (Kapa Biosystems-Roche, Basel, Switzerland), amplified via PCR, quantified via Qubit (Life Technologies, Carlsbad, CA, USA), and then combined into equimolar pools before sequencing. In the latter, libraries were prepared with a Nextera XT Library Prep Kit (Illumina, San Diego, CA, USA) using the standard protocol but with twice the input DNA and 45 additional seconds during PCR elongation prior to quantification. In each case, samples were sequenced using 250 bp paired-end reads on an Illumina MiSeq instrument. Reads were trimmed using Trimmomatic 0.36 (5) with a sliding window quality cutoff of 15 and assembled *de novo* using SPAdes 3.7 (6) either via MicrobesNG or locally via the same methods. Assembly quality was determined using CHECKM v.1.2.2 (7), and the genomes were annotated using the NCBI Prokaryotic Genome Annotation Pipeline Build 6771 (8) (based on PGAP 6.5) or Prokka 1.11 (9). Contamination was identified and removed using ContEst16 (10) and the automated NCBI Foreign Contamination Screen 5.4 (11). Lastly, we used HaloClass 1.0 (12), a state-of-the-art protein classifier, to determine the percentage of the annotated proteins in each sample that were likely halophilic in comparison to an *Escherichia coli* K12 control sample. The default parameters were applied for all programs.

Seven draft genomes were assembled and submitted to the NCBI’s genome database. The mean N50 value was 502,298 bp, and the mean L50 value was 5.7, calculated using the R package BioStrings 3.21 (13). Taxonomy was estimated using BLAST 2.16.0, with 16S percent identities listed (14) (Table 1). Contigs smaller than 500 bp were removed prior to submission. We identified four genera of bacteria (*Halomonas*, *Halobacillus*, *Staphylococcus*, and *Thalossobacillus*). We found that all of our genomes were predicted to have substantially more distinct salt-tolerant proteins than our *E. coli* K12 control (30.8%–4.2% salt-tolerant proteome compared to 1.1% in *E. coli* K1, Table 1). Genome coverage data and additional metadata are available in the BioSample and Genome attributes on the NCBI database.

## Data Availability

All seven whole-genome sequences have been deposited to the NCBI’s genome database under BioProjects PRJNA1164789 and PRJNA1169026.

## References

[1] Madern D, Ebel C, Zaccai G. 2000. Halophilic adaptation of enzymes. Extremophiles 4:91–98. doi:10.1007/s007920050142 10805563

[2] Nguyen PT, Nguyen-Thi TU, Nguyen HT, Pham MN, Nguyen TT. 2024. Halophilic lactic acid bacteria — Play a vital role in the fermented food industry. Folia Microbiol 69:305–321. doi:10.1007/s12223-024-01149-0 38372951

[3] Liu C, Baffoe DK, Zhan Y, Zhang M, Li Y, Zhang G. 2019. Halophile, an essential platform for bioproduction. J Microbiol Methods 166:105704. doi:10.1016/j.mimet.2019.105704 31494180

[4] Khanafari A, Khavarinejad D, Mashinchian A. 2010. Solar salt lake as natural environmental source for extraction halophilic pigments. Iran J Microbiol 2:103–109. 22347558 PMC3279771

[5] Bolger AM, Lohse M, Usadel B. 2014. Trimmomatic: a flexible trimmer for Illumina sequence data. Bioinformatics 30:2114–2120. doi:10.1093/bioinformatics/btu170 24695404 PMC4103590

[6] Bankevich A, Nurk S, Antipov D, Gurevich AA, Dvorkin M, Kulikov AS, Lesin VM, Nikolenko SI, Pham S, Prjibelski AD, Pyshkin AV, Sirotkin AV, Vyahhi N, Tesler G, Alekseyev MA, Pevzner PA. 2012. SPAdes: a new genome assembly algorithm and its applications to single-cell sequencing. J Comput Biol 19:455–477. doi:10.1089/cmb.2012.0021 22506599 PMC3342519

[7] Parks DH, Imelfort M, Skennerton CT, Hugenholtz P, Tyson GW. 2015. CheckM: assessing the quality of microbial genomes recovered from isolates, single cells, and metagenomes. Genome Res 25:1043–1055. doi:10.1101/gr.186072.114 25977477 PMC4484387

[8] Tatusova T, DiCuccio M, Badretdin A, Chetvernin V, Nawrocki EP, Zaslavsky L, Lomsadze A, Pruitt KD, Borodovsky M, Ostell J. 2016. NCBI prokaryotic genome annotation pipeline. Nucleic Acids Res 44:6614–6624. doi:10.1093/nar/gkw569 27342282 PMC5001611

[9] Seemann T. 2014. Prokka: rapid prokaryotic genome annotation. Bioinformatics 30:2068–2069. doi:10.1093/bioinformatics/btu153 24642063

[10] Lee I, Chalita M, Ha SM, Na SI, Yoon SH, Chun J. 2017. ContEst16S: an algorithm that identifies contaminated prokaryotic genomes using 16S RNA gene sequences. Int J Syst Evol Microbiol 67:2053–2057. doi:10.1099/ijsem.0.001872 28639931

[11] Astashyn A, Tvedte ES, Sweeney D, Sapojnikov V, Bouk N, Joukov V, Mozes E, Strope PK, Sylla PM, Wagner L, Bidwell SL, Brown LC, Clark K, Davis EW, Smith-White B, Hlavina W, Pruitt KD, Schneider VA, Murphy TD. 2024. Rapid and sensitive detection of genome contamination at scale with FCS-GX. Genome Biol 25:60. doi:10.1186/s13059-024-03198-7 38409096 PMC10898089

[12] Narang K, Nath A, Hemstrom W, Chu SKS. 2024. HaloClass: salt-tolerant protein classification with protein language models. Protein J 43:1035–1044. doi:10.1007/s10930-024-10236-7 39432175 PMC11543744

[13] Pagès H, Aboyoun P, Gentleman R, DebRoy S. 2025. Biostrings: Efficient manipulation of biological strings. R package version 3.21.0. Bioconductor. https://bioconductor.org/packages/Biostrings.

[14] Altschul SF, Gish W, Miller W, Myers EW, Lipman DJ. 1990. Basic local alignment search tool. J Mol Biol 215:403–410. doi:10.1016/S0022-2836(05)80360-2 2231712

